# The Early Shorebird Will Catch Fewer Invertebrates on Trampled Sandy Beaches

**DOI:** 10.1371/journal.pone.0161905

**Published:** 2016-08-26

**Authors:** Thomas A. Schlacher, Lucy K. Carracher, Nicholas Porch, Rod M. Connolly, Andrew D. Olds, Ben L. Gilby, Kasun B. Ekanayake, Brooke Maslo, Michael A. Weston

**Affiliations:** 1 School of Science and Engineering, The University of the Sunshine Coast, Q-4558, Maroochydore, Australia; 2 Centre for Integrative Ecology, School of Life and Environmental, Deakin University, Geelong, Australia; 3 Australian Rivers Institute - Coast & Estuaries, and School of Environment, Gold Coast Campus, Griffith University, Southport, Australia; 4 Department of Ecology, Evolution and Natural Resources, Rutgers, The State University of New Jersey, 55 Commercial Ave, New Brunswick, NJ, 08901, United States of America; University of Fribourg, SWITZERLAND

## Abstract

Many species of birds breeding on ocean beaches and in coastal dunes are of global conservation concern. Most of these species rely on invertebrates (e.g. insects, small crustaceans) as an irreplaceable food source, foraging primarily around the strandline on the upper beach near the dunes. Sandy beaches are also prime sites for human recreation, which impacts these food resources via negative trampling effects. We quantified acute trampling impacts on assemblages of upper shore invertebrates in a controlled experiment over a range of foot traffic intensities (up to 56 steps per square metre) on a temperate beach in Victoria, Australia. Trampling significantly altered assemblage structure (species composition and density) and was correlated with significant declines in invertebrate abundance and species richness. Trampling effects were strongest for rare species. In heavily trafficked plots the abundance of sand hoppers (Amphipoda), a principal prey item of threatened Hooded Plovers breeding on this beach, was halved. In contrast to the consistently strong effects of trampling, natural habitat attributes (e.g. sediment grain size, compactness) were much less influential predictors. If acute suppression of invertebrates caused by trampling, as demonstrated here, is more widespread on beaches it may constitute a significant threat to endangered vertebrates reliant on these invertebrates. This calls for a re-thinking of conservation actions by considering active management of food resources, possibly through enhancement of wrack or direct augmentation of prey items to breeding territories.

## Introduction

A bird came down the walk:

He did not know I saw;

He bit an angle-worm in halves

And ate the fellow, raw.

“A Bird Came Down” Emily Dickinson

Globally, ocean beaches attract humans whose activities cause numerous detrimental impacts to beach ecosystems [1–3]. Beaches are prime sites for recreation[4]. From an environmental perspective, one of the ways that recreation manifests itself is through the impact of trampling by people, a near-ubiquitous impact which is most severe on developed coasts. The harmful consequences of trampling are well documented for coastal dunes, especially the impacts on vegetation [4]. For the non-vegetated part of the shore, seawards of the dunes, trampling impacts are either insufficiently quantified or are confounded with more diffuse pressures from urbanization, shore armouring, or grooming [5, 6]. The two studies that could attribute changes in shallow-buried beach invertebrates to human trampling show declines in abundance for species of the middle and lower shore [7, 8]. It is, however, largely unknown whether—and to which extent—trampling may impact invertebrates on the *upper* shore where many shorebirds feed and human foot traffic is concentrated during high tides [9].

Ocean beaches are habitat for diverse assemblages of invertebrates [10, 11]. On the upper shore near the dunes, these assemblages are mainly composed of insects (e.g. coleoptera, diptera) and smaller peracarid crustaceans (e.g. amphipods, isopods) that are buried in the sand or under wrack during the day and become surface active at night [12]. Beach invertebrates are an irreplaceable food source for beach-nesting birds, a group that contains several species of critical conservation concern [13]. Obligate beach-nesting birds depend on adequate invertebrate prey, particularly in the upper beach [14], actively select prey-rich sections of beaches [15], and have energetically-demanding reproductive strategies [16]. Given that food is often a limiting resource and that many shorebirds are in decline worldwide, processes that diminish prey abundance are likely to be of major importance to the conservation of beach-dwelling birds [9].

**Fig 1:**
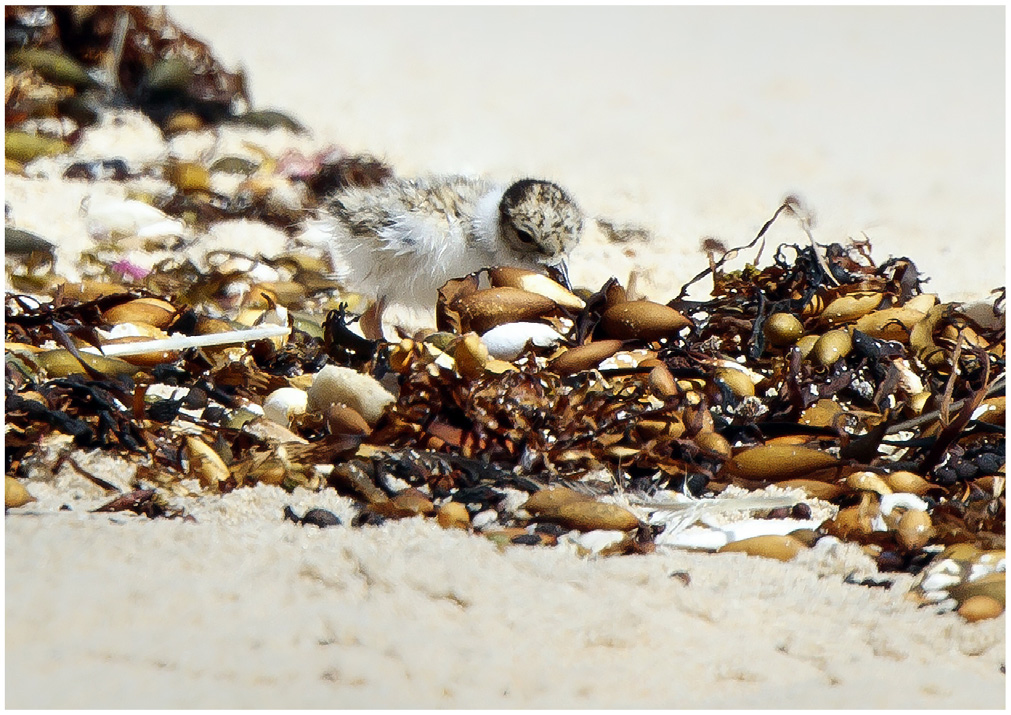
A hooded plover chick feeding on the strandline of beaches in the study region (Photo: Glenn Ehmke).

Plovers on ocean beaches, worldwide rely heavily on upper beach invertebrates as a food source (Fig 1). They have catholic tastes, consuming a diversity of prey items that generally includes amphipods, isopods, molluscs, worms and a broad range of insects (e.g. beetles, dipterans, ants) [17–19]. Plovers regularly forage across habitat boundaries, feeding on exposed shores, dunes and upland wetlands during the day and at night [20]. In hooded plovers, biparental care and low reproductive success also mean that adults have limited mobility for long periods when breeding (incubation: 30 days; brood-rearing: 35 days) and that breeding territories are used for up to eight months per year [21]. Thus, food availability is a pivotal factor in habitat selection in beach-associated birds, extending also to non-breeding periods [15, 19, 22, 23].

Management interventions for threatened beach birds usually encompass reducing disturbance and direct physical threats through access restrictions, predator control, and provisioning of shelter [24, 25]. By contrast, management interventions focusing on the conservation and supplementation of food resources for obligate beach birds are rarely implemented. Trampling might reduce the abundance of prey available to beach-foraging birds and impact on the conservation of these species, but this hypothesis has rarely been tested with empirical data. “Hooded Plovers often breed in areas heavily used by recreationists [26] and their habitat is often heavily trampled (M.A. Weston pers. obs.).” Thus, here we examine whether intense human trampling acutely alters upper-shore invertebrate populations on a beach that supports a breeding population of the threatened Hooded Plover, *Thinornis rubricollis*.

## Methods

### Ethics Statement

All field work was undertaken in in accordance with Permit No.10007183 issued for the work by the Victorian Department of Primary Industry and Environment. It did not involve the use of animals requiring animal ethics committee clearance by Deakin University or any other body with jurisdiction for the site or the taxa. In no instance was research undertaken on private land. No other permissions were necessary for the research reported here.

### Study Site

We ran an experiment to determine the acute effects of human trampling on upper-shore invertebrates on an exposed (mean wave height 1.8 m) beach on the south coast of Australia. The experimental site was Venus Bay (-38.71°, 145.82°) in Victoria, 170 km south of Melbourne. The beach is ca. 30 km long, faces south-west, and is of the intermediate morphodynamic type (100–150 m wide intertidal zone at low tide that slopes relatively gently (3–8°) and is composed of sands with a median grain size of 200–260 microns). The site was chosen for two reasons: 1) it represents important breeding habitat for a sandy-shore obligate threatened species, the Hooded Plover, a species for which human trampling may detrimentally affect prey resources; and, 2) some beach sections have very few visitors (M. Weston pers. obs.) and hence had low human trampling impacts before the experiment.

### Layout of Field Experiments

**Fig 2:**
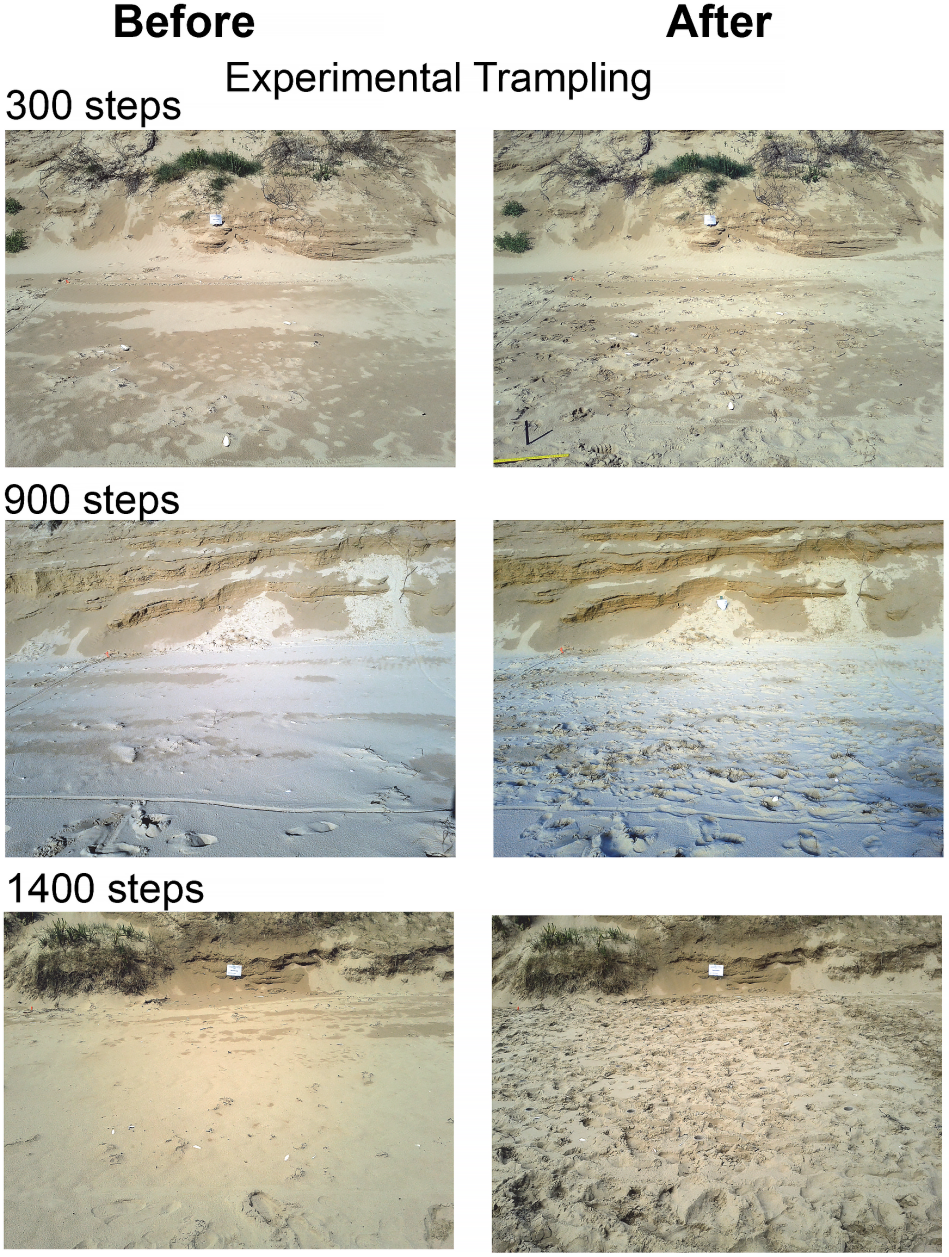
Illustration of plot lay-outs and surface disturbance caused by experimental human trampling.

We dispersed fourteen 5 x 5-m plots (Fig 2) along the upper part of the shore between the last high-tide mark and the base of the dunes using four criteria: 1) maximize the distance from beach access points to avoid interference from beach visitors; 2) avoid known nest sites; 3) exclude very narrow sections of the beach where tides would likely inundate experimental plots; and 4) separate adjoining plots by at least 50 m alongshore (the actual mean distance between plots was 233 m).

We randomly assigned a trampling intensity to each experimental plot, which ranged between 0 and 1,400 steps, applied at increments of 100. We effected trampling by allowing three-person teams (weight 55–80 kg) to walk barefoot on the beach inside the plots until the required number of steps was reached. The treatment was applied such that an approximately even spread of footprints occurred across the plot; this was achieved by having an observer directing the walkers to “spread out” the trampling and who was aware how many steps remained. The experiment was done on 8 Dec. 2014. Ethical considerations demanded that we limited any physical alterations to the beach habitat and avoided disturbing nesting birds; hence, all experimental trampling was limited to a maximum intensity of 1,400 steps per plot and to a single pulse event. While few studies of trampling on beaches report density of steps for prevailing recreational trampling, our trampling rates visually appear typical of heavy recreational use (Fig 2). The density of steps was, however, less than that associated with spatially constrained sports activities (i.e. volleyball).

### Sampling

Surface-active invertebrates were sampled with pitfall traps (350 ml plastic cups, 75 mm diameter, 110 mm deep, half filled with a mix of seawater and detergent, sunk into the sand so the rim was flush with the surface). We placed 25 traps, separated by 1 m, within each plot. Traps were placed after trampling and in a manner so as not to alter trampling rates (i.e. from the edge and from a board positioned so that it enabled access to the centre of the plot).

Traps were placed immediately after the trampling treatment near sunset and left out overnight, with a deployment duration of of 851 ± 9 (se) minutes; Cuttris et al. [23] showed that on very similar beaches in the same region, species saturation in pitfall collections is reached after approximately 360 minutes.

Sediment compactness was measured by dropping a stainless steel rod (weight 450 g, diameter 10 mm, point blunt) five times before trampling and averaging the penetration distance. We visually estimated the percentage of plots covered with macroalgal wrack and ca. 100 g of sand was collected from the top 10 cm of each plot for grain size analysis.

### Data Analysis

The chief question was how important foot trampling by walkers is in comparison to habitat features frequently reported to be associated with variations in invertebrate assemblages on upper parts of sandy beaches (i. e. wrack, sediment compactness, grain size). We addressed this with distance-based linear models, DISTLMs, [27], relating trampling intensity, deployment duration of traps and environmental predictors to three complementary fauna metrics (assemblage structure, abundance, species richness). For the fauna, similarity matrices were based on Bray-Curtis resemblances (calculated from untransformed abundance data) for multivariate assemblage structure n and Euclidean distances for total catch (individuals per trap, untransformed) and species richness (number of species per trap). All analyses were run on data pooled over all traps per plot, using the PRIMER software [28]. The contribution of individual species to the total dissimilarity between reference and trampled plots was examined with the SIMPER (similarity percentages) of Primer [28].

Model performance was evaluated using the corrected Akaike Information Criterion (AICc) based on all possible combinations of variables used in model building [29, 30]. A multi-model inference approach was employed to assess the contributions of individual variables based on their summed Akaike weights [31]: summed AICc weights (w+) provide relative probabilities of variable importance, with variables < 0.3 likely to be of minor or no importance [32]. As a diagnostic before the model runs, we checked for confounding between experimental treatments and habitat attributes and found that trampling intensity was not significantly correlated with any of the environmental variables (max. r = 0.47; min. P = 0.09).

## Results

The intensity of experimental trampling was the most influential predictor for observed heterogeneity in invertebrate assemblages (Table 1, S1 Table). Trampling was the highest-ranked predictor in all models, based on either variable weights or the proportion of variance accounted for; no other tested variable accounted for significant fractions of variance in assemblage composition or total catch in our models, nor could they be considered important based on their relatively low variable weights (i.e. mostly <0.30; Table 1). The cover of macroalgal wrack that was naturally present on experimental plots was an important predictor of species richness, with a minor contribution from deployment duration (Table 1).

**Table 1:** Contributions of variables in models relating three metrics of invertebrate assemblages (assemblage structure, total catch of individuals, species richness) to experimental trampling, key habitat attributes (wrack cover, sediment compactness, grain size) and the time pitfall traps were deployed during the experiments. Variable contributions are assessed in two complementary ways: i) a multi-model inference approach using cumulative weights, w+(j), and ii) the proportion of variance explained in distance-based linear models.

	Variable Weights w+(j)						Proportion of Variance Explained					
Variable	Assemblage Structure		Catch		No Species		Assemblage Structure		Catch		No. Species	
Trampling	0.67	#	0.77	#	0.99	#	0.26	**	0.34	*	0.54	**
Wrack Cover	0.31		0.39		0.97	#	0.10		0.20		0.42	*
Deployment Duration	0.34		0.20		0.27		0.13		0.13		0.40	*
Sediment Compactness	0.32		0.15		0.19		0.10		0.00		0.00	
Grain Size	0.20		0.24		0.12		0.05		0.11		0.14	

* P < 0.05,

** P < 0.01; marginal tests in DISTLM

^#^ included in best overall model based on lowest AICc value

**Fig 3:**
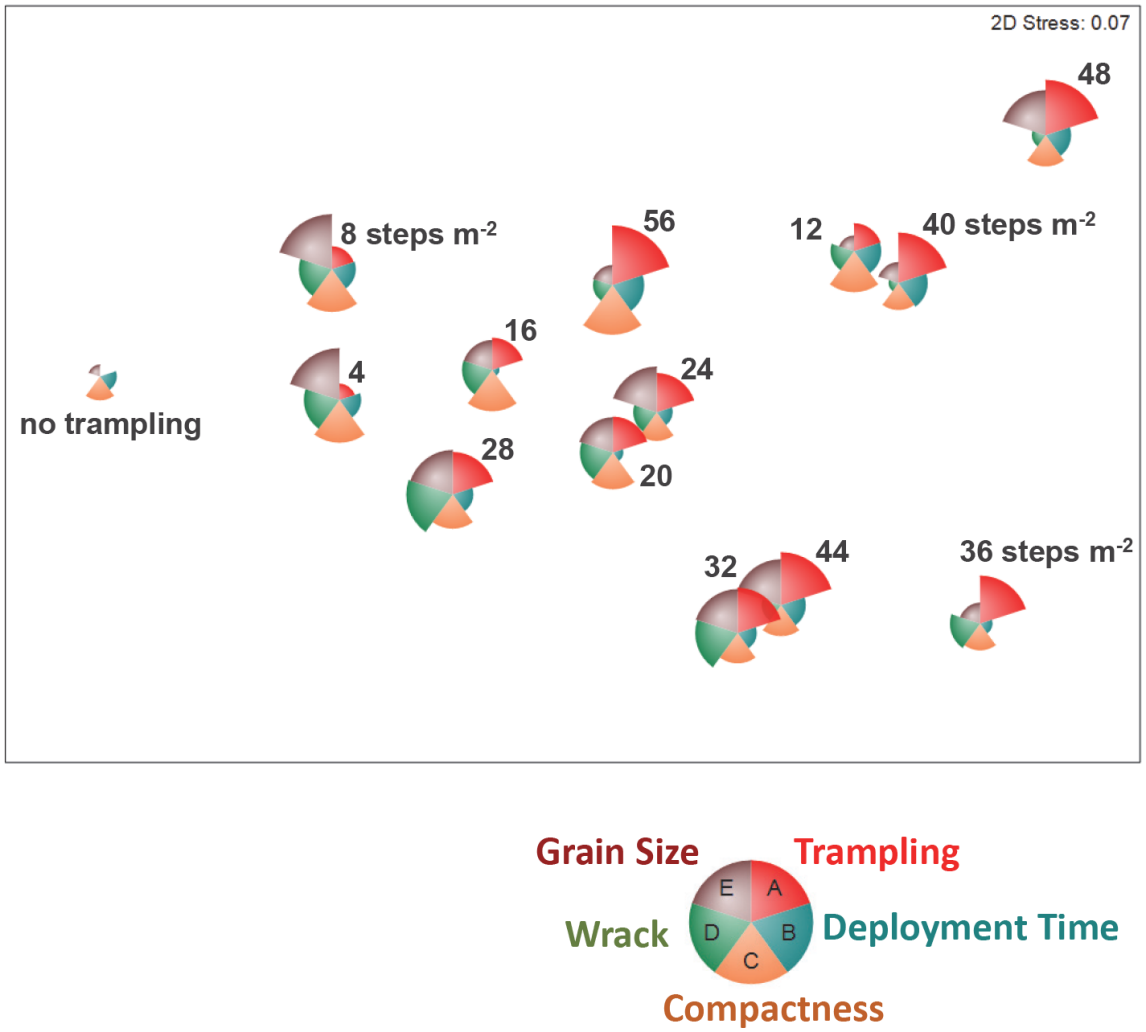
Ordination (non-metric multidimensional scaling) of experimental plots based on similarity (Bray Curtis) in assemblage structure (i.e. species composition and abundance of species) of surface-active invertebrates. Distance between plot symbols is scaled to similarity (i.e. nearby samples have more similar invertebrate catches) and the size of segments is proportional to the value of an environmental or experimental variable and illustrates relative influence (cf. Table 1 for actual model statistics).

**Fig 4:**
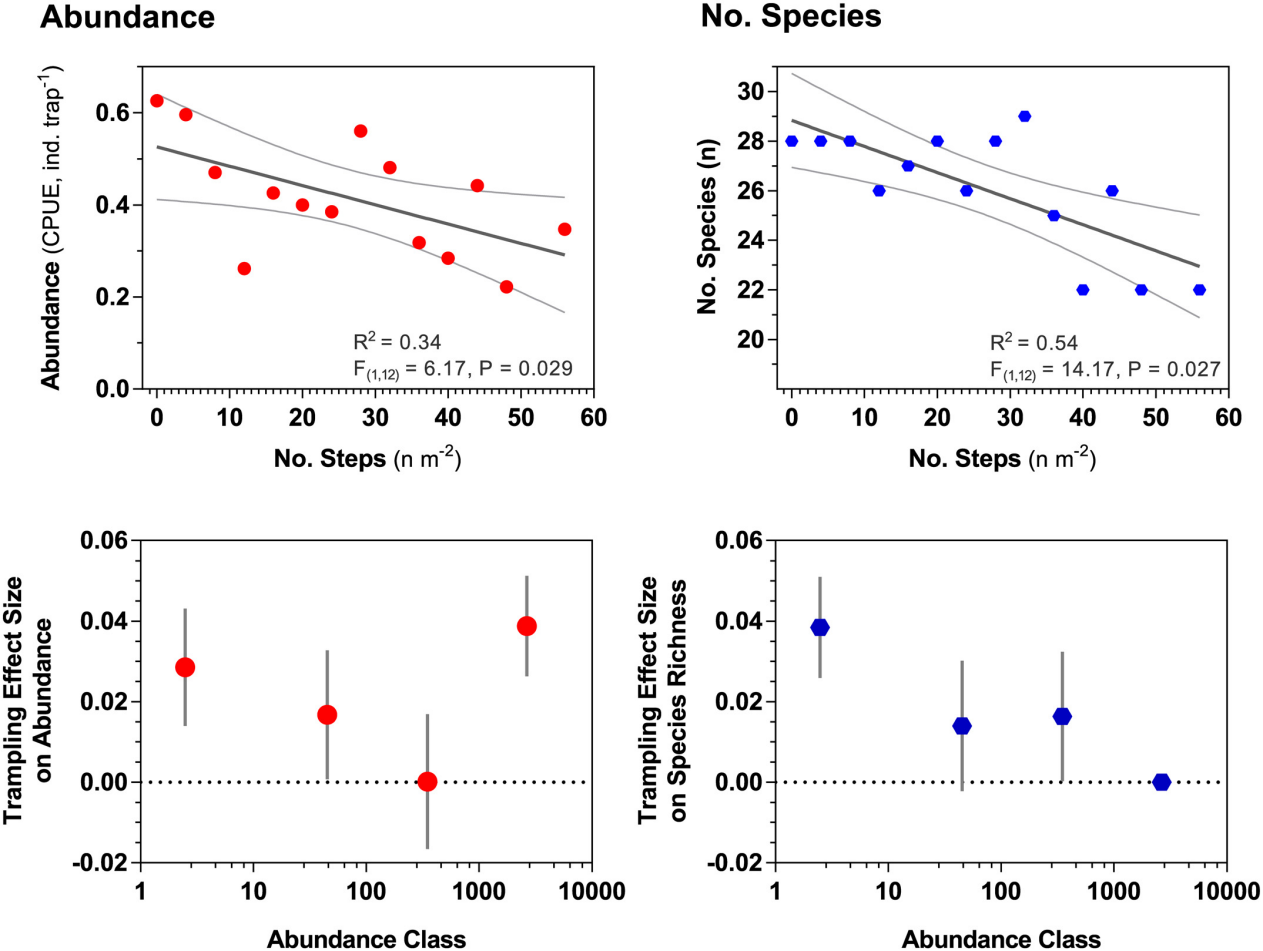
Changes in abundance (left column) and species richness (right column) of surface-active invertebrates in relation to experimental trampling on the upper part of an ocean-exposed beach (statistics inside top panels are for linear regression analysis). Bottom row illustrates trampling effect sizes (indexed as the slope (+/- se) of linear regressions of abundance/species richness vs the number of steps) for logarithmic abundance classes of species.

Experimental trampling was strongly linked to variations in assemblage structure of surface-active invertebrates, and substantially reduced both the size and diversity of catches (Figs 3 and 4). In plots that were not, or lightly, trampled we caught 28 species, declining to 22 species under more intense (> 50 steps m^-2^) foot traffic (Fig 4). Mean density of all species declined by half over the range of experimental trampling applied (Fig 4). Negative effects of trampling on invertebrate abundance were most pronounced for rare species that declined at significantly greater rates in trampled plots: many taxa that occurred at initially low numbers were not found in the more heavily trampled plots. Effect sizes for declines in species richness were highest for the least and the most abundant species (Fig 4).

Nearly half of the shift in assemblage structure observed under experimental trampling was driven by sizeable (-2.4 x) reductions in the density of the talitrid amphipod *Bellorchestia* sp. 1 (Table 2). All other taxa occurred at considerably lower abundance and accounted for comparatively smaller proportions of assemblage change: three species of beetles, an oniscoid isopod, ants, dipterans, a second species of talitrid amphipod and beetle larvae each accounted for > 1% of dissimilarity in assemblage structure observed between the trampled and the control plots (Table 2).

**Table 2:** Summary of similarity percentage analysis (SIMPER) listing species that cumulatively contributed 90% to the dissimilarity (Bray Curtis) in community structure between trampled and un-trampled plots.

Species (Higher Taxon)	Control—Mean Abundance (ind. trap^-1^)	Trampled Mean Abundance (ind. trap^-1^)	Avg. Dissimilarity	Diss / SD	Contribution (%)
*Bellorchestia* sp.1 (Amphipoda, Talitridae)	23.00	9.52	21.79	2.11	46.45
*Phycosecis litoralis* (Coleoptera-Phycosecidae)	1.25	5.01	5.73	2.99	12.21
Diptera sp.3	3.88	0.99	4.57	2.75	9.73
*Sartallus signatus* (Coleoptera, Staphylinidae)	3.00	1.51	2.47	1.83	5.25
Lycosid spider sp.1 (Araneae, Lycosidae)	0.38	1.72	2.16	0.61	4.60
Oxyteline cf. Carpelimus sp.1 (Coleoptera, Staphylinidae)	1.75	0.40	2.10	5.89	4.48
*Actaecia thomsoni* (Isopoda, Actaeciidae)	2.08	1.24	1.37	1.75	2.92
*Phycosecis spp*. *Larvae* (Coleoptera-Phycosecidae)	0.42	1.25	1.30	1.53	2.77
*Bellorchestia* sp.2 (Amphipoda, Talitridae)	0.46	0.84	0.66	0.99	1.41
Ant sp.1 (Hymenoptera, Formicidae)	0.50	0.08	0.66	5.38	1.41

## Discussion

Anthropogenic habitat change resulting from recreational activities is widespread on sandy coastlines [4]. Here, the ecological consequences of disturbance caused by vehicles and pedestrian trampling are extensive and well-documented for dunes and their biota [33]. By contrast, on the non-vegetated part of the shore, only impacts caused by vehicles have been comprehensively quantified whereas the effects of foot traffic are generally more sparsely covered [34–36]. We addressed this gap with a high-intensity, but short-term, experiment demonstrating acute ecological effects resulting from beach walkers. Detrimental ecological effects of physical impacts from vehicles are generally manifested over large areas and are persistent [37, 38]. By comparison, trampling effects can, on lightly used beaches, be more localized and transient [35], but lead to persistent reductions in invertebrates on more intensively used shores [5, 33, 35, 39]. Thus, whether trampling will have negative consequences for shorebirds will largely depend on the intensity of human foot traffic, particularly during the breeding season.

Our focal species, the Hooded Plover, feeds extensively on the invertebrates observed to be most heavily impacted by trampling in our experiment. When feeding on ocean beaches, these plovers use both darting and foot-trembling as foraging tactics, and regularly chase and consume surface-active talitrid amphipods [14]. Importantly, sand hoppers constituted 43% of all individuals caught in our experiment and the abundance of this key prey item declined substantially with foot traffic (from a total catch of 563 individuals in the reference plot to 260 individuals over the range of trampling intensities tested). Thus, human foot traffic reduced, at least in the short-term, the main food source of a threatened bird species, which is the target for considerable conservation investment.

Because energy requirements in birds are particularly high during nesting and brood-rearing, there are two plausible consequences of trampling-induced declines in food availability. At the level of nesting pairs, young-of-the-year birds could experience lower fitness. The precocial chicks of beach-nesting birds are flightless and require access to abundant local food resources to promote rapid growth and development [40]. Those not acquiring adequate resources in this critical growth phase experience poor first-year survival [41][42]. At the population level, trampling effects may exacerbate existing density-dependent effects of food availability and ultimately reduce overall carrying capacity of target species. Solitary beach-nesting bird species can exhibit a clumped dispersion where food resources are abundant. In these areas, territory size is small because adults can secure enough resources for the brood within a localized area. Where prey availability is limited, however, competition and territory size increase, reducing the number of pairs using a given habitat. At carrying capacity, there can be no sustained increase in population size, as survival and reproductive success are severely constrained.

The ‘*standard repertoire*’ of beach-bird conservation usually encompasses any combination of the following five actions: i) restrict access (seasonal or spatial beach closures), ii) educate beach users (etc. signs, awareness campaigns), iii) control predators; iv) provide extra shelter; and v) enhance or restore habitat [24, 43]. These interventions generally have mixed success, chiefly because invasive species control is incomplete (save for small islands) and because compliance of humans with control measures or environmental messages can be low [44–47]. Our results demonstrate that human trampling might be a cryptic factor that limits the success of beach-nesting bird conservation, and highlight the need for fresh approaches to management. We propose two actions that may partially offset the negative impacts of trampling on threatened beach-nesting birds: (1) wrack relocation; and (2) invertebrate augmentation. Wrack supports invertebrate prey for birds through the creation of habitat and the supply of food [19, 48]. As a component of recreational management on beaches, wrack is often removed from beaches and discarded. These spoils may, however, be deposited in or near breeding territories of plovers (following appropriate protocols) to enhance invertebrate food resources. Wrack relocation and deposition could have the added benefit of reducing disturbance to breeding birds because walkers may be deterred by wrack piles placed strategically near nests.

An alternative strategy for locations where trampling is very intense and the supply of wrack is limited could be to directly supplement invertebrate prey to the breeding territories of beach-nesting birds. Many plovers on ocean beaches have high site fidelity and limited movement [19, 23]. Theoretically, it may therefore be possible to obtain invertebrate prey by harvesting limited numbers of individuals from sites that are consistently not used by plovers and augment these to existing breeding territories. Before this is considered as a conservation tool, it needs to be demonstrated that this action does not significantly deplete invertebrate populations at harvest sites and that any augmented prey will be available to plovers for a meaningful periods of time. Furthermore, whether this method is cost-effective and practicable needs to be tested. It is critically important to note that neither wrack relocation nor invertebrate augmentation are likely to replace more traditional approaches that restrict access and provide nest shelter, but both techniques can be explored as a useful complement, particularly for beaches that support prime breeding habitat and those where human compliance with regulations is particularly poor.

The ecological impacts of foot traffic on ocean beaches will in most situations depend on a combination of: a) the number of people traversing a beach (physical intensity), b) the types of activities undertaken (e.g. walking or games); c) the frequency and temporal mode at which trampling occurs (e.g. pulsed, dispersed); and d) the time of the year especially with regards to the breeding season of species sensitive to disturbance. Furthermore, the action of trampling is usually not conducted in isolation from other human pressures on beaches, and in many cases it is accompanied by direct disturbance from humans, at times amplified by their companion dogs or vehicles used to travel on beaches [22, 44, 49–51]. Thus, trampling can exert at least three modes of pressure on beach birds: i) declines in food resources (crushing of invertebrates), direct disturbance by humans (investment in escape and alter behaviour), and disturbance impacts from vehicles and dogs [52, 53]. All these factors are amenable to specific experimental tests in the future. Experiments will be particularly useful in situations where certain information is required to inform conservation decisions (e.g. thresholds of impact) or where potential management scenarios are to be assessed (e.g. temporal or spatial restrictions, number of walkers etc.). A major challenge for experiments that seek to quantify the magnitude of human impacts is to ensure that additional environmental harm is kept to a minimum.

Here we have shown that trampling effects on beaches can limit food availability for beach-nesting birds, which in turn may reduce the carrying capacity of beaches and lead to increased mortality of nesting individuals and their offspring. It will, therefore, be critical to identify the thresholds of pedestrian trampling beyond which declines in invertebrates become permanent and propagate to detrimental effects on threatened birds more broadly.

## Supporting Information

S1 Table Containing complete taxa x plot data. (PDF)
