# Supplementary material for: The Early Shorebird Will Catch Fewer Invertebrates on Trampled Sandy Beaches
Source: PLoS One. 2016 Aug 26;11(8):e0161905. doi: 10.1371/journal.pone.0161905 (PMC5001726; doi:10.1371/journal.pone.0161905)
Supplement: S1 Table — (PDF) [file pone.0161905.s001.pdf]

S1, Table

Schlacher et al: *The early shorebird will catch fewer invertebrates on trampled sandy beaches.*

Supporting Information

	2	3	4	5	6	7	8	9	10	11	12	13	15	16
Amphipoda-Talitridae-Bellorchestia_sp.	9.08	3.88	5.08	2.64	8.00	18.92	19.08	13.50	7.12	8.84	4.04	10.08	13.48	23.00
Amphipoda-Talitridae-amphsp2	0.12	0.84	0.76	1.32	0.44	0.72	0.88	2.13	1.40	0.56	0.56	0.68	0.48	0.46
Araneae-aranfam1-aransp4	0.00	0.04	0.00	0.00	0.00	0.00	0.00	0.00	0.00	0.00	0.00	0.00	0.00	0.04
Araneae-aranfam2-aransp7	0.00	0.04	0.04	0.00	0.00	0.00	0.00	0.00	0.00	0.04	0.00	0.00	0.04	0.00
Araneae-aranfam3-aransp3	0.00	0.00	0.04	0.00	0.00	0.04	0.00	0.00	0.00	0.00	0.00	0.04	0.00	0.00
Araneae-isofam1-aransp1	0.04	0.00	0.00	0.00	0.00	0.00	0.00	0.00	0.00	0.00	0.00	0.00	0.00	0.00
Araneae-lycosidae-aransp1	0.36	0.28	0.24	0.36	6.68	0.48	0.27	1.17	6.20	0.48	4.72	0.88	0.20	0.38
Araneae-lycosidae-aransp8	0.00	0.00	0.00	0.00	0.00	0.00	0.00	0.00	0.00	0.00	0.00	0.04	0.00	0.00
blattodea-blattidae-blatsp1	0.00	0.00	0.00	0.00	0.00	0.00	0.00	0.00	0.00	0.00	0.00	0.04	0.00	0.00
Coleoptera-Anthicidae-Lagrioida australis	0.00	0.00	0.00	0.00	0.00	0.00	0.00	0.00	0.04	0.00	0.00	0.00	0.00	0.00
Coleoptera-anthicidae-mecynotarsus sp.	0.00	0.00	0.00	0.00	0.04	0.04	0.04	0.04	0.16	0.00	0.08	0.08	0.04	0.00
Coleoptera-carabidae-Pterostichine sp.	0.00	0.00	0.00	0.00	0.00	0.04	0.00	0.00	0.00	0.00	0.00	0.00	0.00	0.00
Coleoptera-Carabidae-scaraphites rotundipennis	0.00	0.00	0.08	0.04	0.00	0.00	0.04	0.00	0.00	0.00	0.00	0.08	0.04	0.04
Coleoptera-chrysomelidae-colsp17	0.00	0.00	0.00	0.00	0.00	0.00	0.00	0.00	0.00	0.12	0.08	0.16	0.00	0.00
Coleoptera-curculionidae-Adelognathid sp.	0.00	0.00	0.00	0.00	0.04	0.04	0.00	0.00	0.00	0.00	0.00	0.00	0.00	0.00
Coleoptera-curculionidae-cf. Perperus sp.1	0.00	0.00	0.04	0.00	0.00	0.00	0.00	0.00	0.04	0.04	0.00	0.04	0.00	0.00
Coleoptera-curculionidae-colsp25	0.00	0.00	0.00	0.00	0.04	0.00	0.00	0.00	0.00	0.00	0.00	0.00	0.00	0.00
Coleoptera-curculionidae-colsp5	0.00	0.04	0.00	0.00	0.12	0.12	0.08	0.04	0.04	0.12	0.16	0.00	0.00	0.17
Coleoptera-curculionidae-Cryptorhynchine sp.	0.00	0.00	0.00	0.00	0.00	0.00	0.00	0.00	0.00	0.00	0.00	0.00	0.04	0.00
Coleoptera-curculionidae-Leptopius sp.	0.00	0.00	0.00	0.00	0.00	0.00	0.00	0.00	0.00	0.00	0.00	0.00	0.04	0.00
Coleoptera-curculionidae-Mandalotus sp.1	0.00	0.00	0.00	0.00	0.00	0.00	0.00	0.00	0.00	0.00	0.00	0.00	0.04	0.00
Coleoptera-Elateridae-Paracardiophorus sp.	0.00	0.04	0.24	0.04	0.12	0.08	0.12	0.08	0.04	0.08	0.04	0.16	0.04	0.04
Coleoptera-histeridae-colsplp	0.08	0.12	0.00	0.08	0.00	0.00	0.04	0.08	0.00	0.08	0.08	0.00	0.00	0.17
Coleoptera-Latridiidae-Corticaria sp.	0.00	0.00	0.00	0.00	0.00	0.08	0.00	0.00	0.00	0.00	0.00	0.00	0.00	0.00
Coleoptera-Phycosecidae-Phycosecis litoralis	6.56	5.20	4.20	2.56	4.40	3.88	7.04	7.42	6.64	4.04	3.72	4.96	4.56	1.25
Coleoptera-ptiliidae-colsp15	0.00	0.00	0.00	0.00	0.00	0.00	0.00	0.00	0.00	0.04	0.00	0.00	0.00	0.00
Coleoptera-Scarabaeidae-Aphodiinae sp.	0.00	0.00	0.00	0.00	0.00	0.00	0.00	0.00	0.00	0.00	0.00	0.00	0.00	0.04
Coleoptera-Scarabaeidae-Aphodius sp.	0.00	0.00	0.00	0.00	0.00	0.04	0.00	0.00	0.00	0.00	0.00	0.00	0.00	0.00
Coleoptera-Scarabaeidae-Melolonthinae	0.00	0.00	0.00	0.00	0.00	0.00	0.00	0.04	0.00	0.00	0.00	0.00	0.00	0.00
Coleoptera-staphylinidae-Carpelimus sp.1	0.08	0.84	0.44	0.60	0.56	0.88	0.23	0.04	0.28	0.00	0.12	0.00	0.24	0.46
Coleoptera-staphylinidae-Oxyteline cf. Carpelimus	0.16	0.48	0.88	0.40	0.52	0.52	0.12	0.50	0.52	0.44	0.40	0.08	0.20	1.75
Coleoptera-staphylinidae-Sartallus signatus	0.72	2.08	0.68	0.96	1.28	0.76	3.23	2.67	2.00	1.76	0.40	1.32	1.76	3.00
Coleoptera-Tenebrionidae-Caediomorpha heteromena	0.00	0.00	0.00	0.00	0.08	0.00	0.00	0.00	0.00	0.00	0.00	0.00	0.00	0.00
Coleoptera-Tenebrionidae-Hyocis bakewelli	0.00	0.00	0.04	0.00	0.00	0.04	0.04	0.00	0.00	0.00	0.00	0.00	0.00	0.00
Coleoptera-Tenebrionidae-Scymena cf. amphibia	0.12	0.12	0.16	0.04	0.16	0.04	0.54	0.67	0.32	0.32	0.40	0.20	0.32	0.17
Coleoptera-Tenebrionidae-Sphargeris physodes	0.04	0.24	0.24	0.16	0.24	0.32	0.31	0.13	0.36	0.76	0.04	0.24	0.20	0.13
diplopoda-diplofam1-diplosp1	0.00	0.00	0.00	0.00	0.00	0.04	0.00	0.00	0.04	0.00	0.00	0.04	0.00	0.00
diplopoda-diplofam2-diplosp2	0.04	0.00	0.00	0.00	0.00	0.00	0.00	0.04	0.00	0.00	0.00	0.00	0.00	0.00
Diptera-dipfam10-dipsp10	0.00	0.00	0.00	0.00	0.00	0.00	0.00	0.00	0.00	0.00	0.00	0.00	0.00	0.04
diptera-dipfam12-dipsp12	0.00	0.00	0.00	0.00	0.04	0.00	0.04	0.00	0.00	0.00	0.00	0.00	0.00	0.00
Diptera-dipfam13-dipsp13	0.00	0.00	0.00	0.00	0.00	0.00	0.00	0.00	0.04	0.00	0.00	0.00	0.00	0.00
Diptera-dipfam15-dipsp15	0.00	0.00	0.00	0.00	0.00	0.00	0.00	0.00	0.00	0.00	0.00	0.00	0.04	0.00
Diptera-dipfam16-dipsp16	0.00	0.00	0.00	0.00	0.00	0.00	0.00	0.04	0.00	0.00	0.00	0.00	0.00	0.00
diptera-dipfam1-dipsp1	0.04	0.12	0.12	0.12	0.48	0.00	0.23	0.13	0.28	0.64	0.24	0.84	0.20	0.38
diptera-dipfam2-dipsp2	0.00	0.00	0.00	0.00	0.12	0.00	0.00	0.00	0.00	0.00	0.08	0.00	0.00	0.04
Diptera-dipfam3-dipsp3	0.04	0.28	0.04	0.24	0.32	0.00	1.27	1.88	0.80	2.16	2.12	2.04	1.64	3.88
diptera-dipfam4-dipsp4	0.00	0.00	0.00	0.00	0.08	0.00	0.04	0.00	0.00	0.00	0.00	0.00	0.00	0.00
diptera-dipfam5-dipsp5	0.04	0.00	0.08	0.00	0.00	0.00	0.15	0.13	0.04	0.08	0.08	0.04	0.12	0.13
diptera-dipfam8-dipsp8	0.04	0.00	0.04	0.00	0.00	0.00	0.08	0.00	0.00	0.00	0.36	0.16	0.12	0.33
geophilomorpha-chilofam1-chilosp1	0.48	0.00	0.04	0.04	0.08	0.04	0.19	0.04	0.28	0.48	0.32	0.56	0.00	0.00
hemiptera-hemfam1-hemsp1	0.00	0.00	0.00	0.00	0.00	0.00	0.00	0.00	0.00	0.00	0.00	0.00	0.04	0.00
hemiptera-hemfam2-hemsp2	0.00	0.00	0.00	0.00	0.00	0.00	0.00	0.00	0.04	0.00	0.00	0.00	0.00	0.00
hymenoptera-formicidae-formsp1	0.00	0.12	0.00	0.08	0.04	0.08	0.00	0.08	0.16	0.12	0.04	0.12	0.16	0.50
hymenoptera-formicidae-formsp2	0.00	0.00	0.08	0.00	0.00	0.00	0.00	0.00	0.00	0.04	0.00	0.04	0.00	0.00
Hymenoptera-Formicidae-formsp3	0.00	0.00	0.00	0.04	0.00	0.04	0.00	0.00	0.04	0.00	0.00	0.00	0.00	0.00
hymenoptera-formicidae-formsp4	0.00	0.00	0.00	0.00	0.00	0.04	0.00	0.00	0.00	0.00	0.00	0.00	0.00	0.08
Hymenoptera-Formicidae-formsp5	0.00	0.00	0.00	0.00	0.00	0.00	0.04	0.08	0.08	0.00	0.00	0.00	0.00	0.00
Isopoda-isofam1-isosp1	0.16	0.28	0.24	0.36	0.24	0.12	0.12	0.04	0.04	0.32	0.20	0.12	0.16	0.08
Isopoda-isofam2-Actaecia thomsoni	2.28	1.20	1.08	1.84	1.32	0.72	1.73	1.67	0.56	1.00	0.56	0.60	1.52	2.08
Isopoda-isofam3-isosp4	0.00	0.12	0.04	0.04	0.00	0.04	0.00	0.04	0.00	0.04	0.00	0.00	0.00	0.00
larvae-larvfam1-larvsp1	0.80	1.04	1.00	1.80	2.16	0.84	1.50	1.96	2.12	0.76	0.84	1.08	0.32	0.42
larvae-larvfam2-larvsp2	0.52	0.52	0.56	0.24	0.24	0.64	0.04	0.13	0.52	0.88	0.36	0.48	0.80	0.33
larvae-larvfam3-larvsp3	0.08	0.00	0.00	0.00	0.00	0.00	0.08	0.54	0.12	0.00	0.00	0.00	0.00	0.08
Trampling_(Number_steps_per_plot)	1400	1000	300	1200	1100	200	100	700	800	600	900	500	400	0
Deployment_Duration (min)	912	894	881	871	867	862	851	846	839	827	816	811	805	830
Sediment_Compactness_(cm)	5.42	3.00	4.34	3.34	3.32	4.54	4.54	3.62	3.26	3.12	2.98	3.86	4.38	2.78
Wrack_Cover_(%)	2.00	0.50	3.00	1.00	2.00	6.00	7.00	12.00	10.00	3.00	5.00	6.00	5.00	0.00
Grain_Size_(microns)	207.67	208.36	204.61	239.69	241.34	259.33	252.55	238.67	237.72	240.91	208.33	224.20	217.23	202.60
